# Precision rehabilitation for swallowing dysfunction after radiotherapy in head and neck cancer: current evidence, key controversies, and future perspectives

**DOI:** 10.3389/fonc.2026.1732142

**Published:** 2026-02-19

**Authors:** Youwei Li, Hongyun Zheng, Suyan Bi, Rui Zhu, Bo Yuan, Zhonya Li, Tingting Zhao, Wei Zhang

**Affiliations:** 1 Department of Rehabilitation, Yunyang County People’s Hospital, Chongqing, China; 2 Department of Radiotherapy, Yunyang County People’s Hospital, Chongqing, China

**Keywords:** adherence, dysphagia, head and neck neoplasms, precision medicine, quality of life, radiotherapy, rehabilitation, swallowing exercises

## Abstract

**Background:**

Swallowing dysfunction (dysphagia) is a devastating and highly prevalent sequela following radiotherapy (RT) for head and neck cancer (HNC), severely impairing patients’ quality of life and nutritional status. While rehabilitation is the cornerstone of management, the translation of evidence into effective clinical practice is hampered by significant heterogeneity in interventions, conflicting outcomes, and poor adherence.

**Methods:**

This narrative review critically synthesizes current evidence from systematic reviews, randomized controlled trials, and prospective cohort studies published between January 2015 and March 2025. A structured literature search was conducted in the PubMed, Web of science and Embase databases using combinations of keywords including “head and neck neoplasms,” “dysphagia,” “radiotherapy,” “rehabilitation,” “swallowing exercises,” “adherence,” “frailty,” and “precision medicine.” The selection focused on high-impact studies that addressed key challenges, controversies, and emerging paradigms in the field. It moves beyond a descriptive summary to evaluate the contradictions in the literature and propose a framework for precision rehabilitation.

**Results:**

The efficacy of swallowing exercises is well-documented, but critical controversies persist. These include the optimal timing (prophylactic vs. reactive), the superiority of specific exercise regimens, and the unpredictable impact of radiotherapy dose constraints on functional outcomes beyond traditional pharyngeal constrictors. A pivotal, yet often overlooked, factor is patient adherence, which is multifactorial and can be improved through behavioral change techniques and technology-assisted strategies (e.g., mHealth, wearable sensors). Furthermore, emerging evidence highlights the need to consider specific patient phenotypes, such as pre-treatment frailty and the presence of internal lymphedema, which significantly influence rehabilitation success. The integration of objective assessments (e.g., HRM, DIGEST) is crucial for quantifying dysfunction and tailoring interventions.

**Conclusion:**

The field of dysphagia rehabilitation in HNC is evolving from a one-size- fits-all approach towards precision medicine. Future efforts must focus on developing personalized rehabilitation pathways based on individual risk stratification (e.g., frailty, dose to specific musculature), integrating technology for monitoring and motivation, and fostering interdisciplinary collaboration among oncologists, speech-language pathologists, and behavioral scientists to bridge the gap between research evidence and lasting functional recovery.

## 1 Introduction

Head and neck cancer (HNC) represents a significant global health burden. While advancements in multimodal treatments, particularly radiotherapy techniques such as intensity-modulated radiotherapy (IMRT), have substantially improved survival rates (1), they have also led to a growing population of survivors facing long-term functional sequelae (2). Among these, dysphagia (swallowing impairment) stands out as a prevalent and devastating complication, profoundly impacting patient quality of life (QoL), nutritional status, and psychosocial well-being (3, 4).

The clinical and societal burden of dysphagia is considerable. It can lead to debilitating consequences such as aspiration pneumonia, malnutrition, and increased dependency on feeding tubes, which in turn contribute to higher healthcare costs and reduced survival (5, 6). Despite extensive research into preventive and therapeutic strategies, including prophylactic swallowing exercises (7–9), dose optimization to swallowing- related structures (1, 10–13), and novel rehabilitation technologies (14, 15), the evidence base remains marked by significant heterogeneity and inconsistent findings. Several systematic reviews and randomized controlled trials (RCTs) have failed to demonstrate a clear, universal benefit of therapeutic exercises on swallowing safety and efficiency in this population (5, 6, 8, 16). This inconsistency underscores a critical “evidence-to- practice” gap, often attributed to methodological variations, small sample sizes, lack of standardized outcome measures, and, crucially, inadequate attention to patient adherence and individual patient factors (17–20).

Many existing reviews remain descriptive, offering a summary of “what” has been studied but lacking a critical appraisal of “why” results are inconsistent. There is a pressing need to move beyond the simplistic question of “is it effective?” and instead explore “for whom is it effective, under what conditions, and why?” (21). Key moderators of rehabilitation success are emerging from the literature, including pre-treatment frailty (21, 22), the development of internal and external lymphedema (4), specific radiation dose-volume parameters to distinct swallowing muscles (11, 13), and the critical role of adherence, which is itself influenced by behavioral, clinical, and service-delivery factors (17, 19, 23–25).

**Figure 1:**
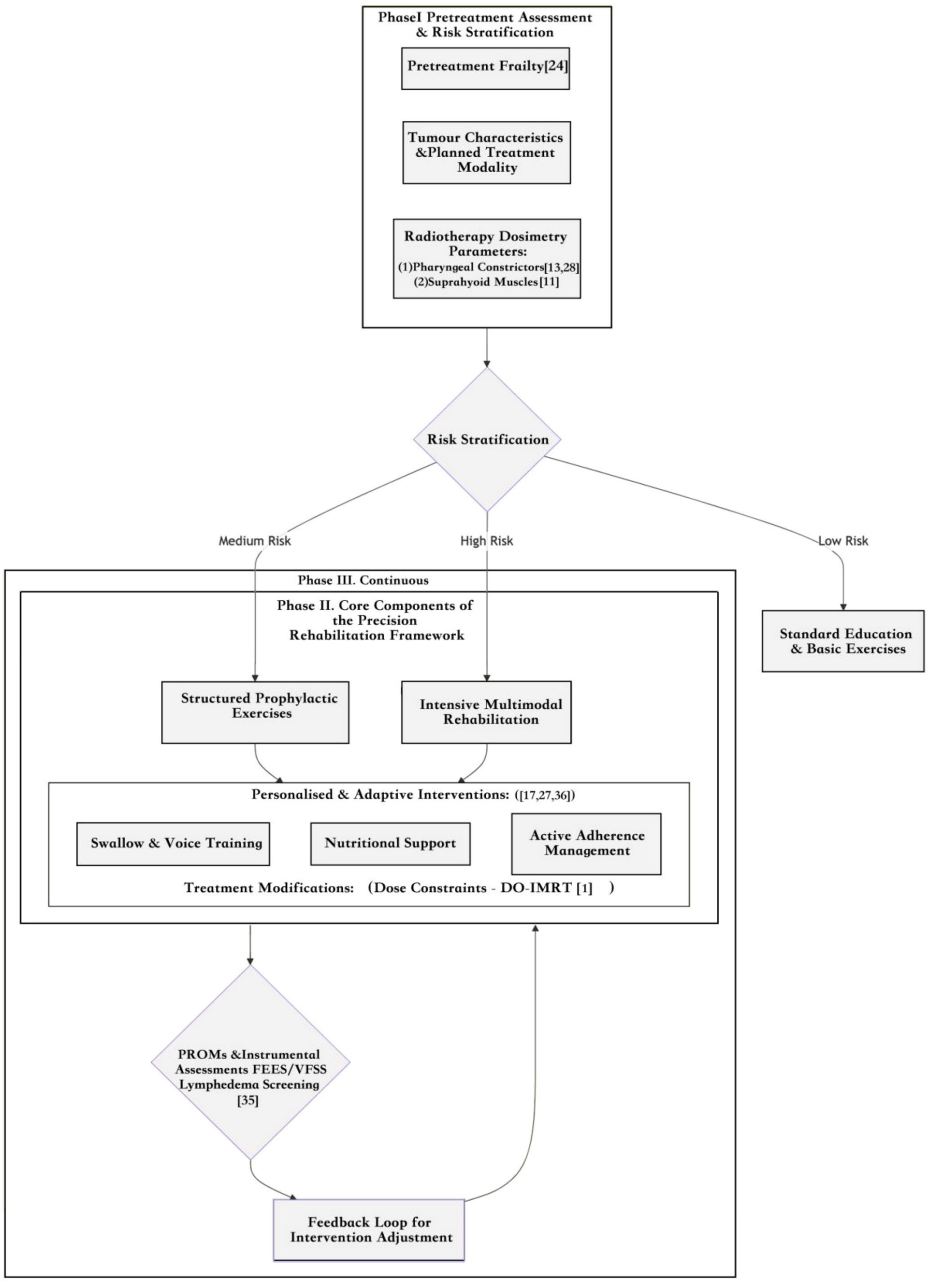
A dynamic precision rehabilitation framework for dysphagia in head and neck cancer. This model illustrates the continuous cycle of assessment, risk stratification, personalized intervention, and monitoring, driven by a central feedback loop for dynamic intervention adjustment. PROMs, Patient-Reported Outcome Measures; FEES, Fiberoptic Endoscopic Evaluation of Swallowing; VFSS, Videofluoroscopic.

This review, therefore, aims to critically synthesize recent high-quality evidence with an explicit focus on bridging this evidence-to-practice gap. To achieve this, high-impact studies published between January 2015 and March 2025 were included based on the following criteria: (1) Study design: randomized controlled trials (RCTs), prospective cohort studies, or systematic reviews; (2) Sample size: ≥50 participants for RCTs or ≥100 participants for cohort studies; (3) Focus: core controversies or innovative paradigms in swallowing rehabilitation following radiotherapy for head and neck cancer (HNC); (4) Publication venue: JCR Q1/Q2 journals in oncology or rehabilitation medicine. Priority was given to highly cited studies (≥50 citations on Google Scholar) and research from authoritative institutions (e.g., MD Anderson Cancer Center, European Head and Neck Cancer Cooperative Group) to ensure the clinical relevance and academic impact of the included evidence. A rigorous quality evaluation process was conducted—employing the GRADE criteria to appraise the certainty of evidence, complemented by domain-specific methodological tools (Cochrane Risk of Bias 2.0 for RCTs, Newcastle-Ottawa Scale for cohort studies, and AMSTAR 2 for systematic reviews) to assess study rigor (26). Notably, all studies incorporated into the subsequent discussion met moderate-to-high quality standards, with no high-risk bias identified in key outcome measures, thus ensuring the reliability of the conclusions presented herein. Based on these studies, we propose and adopt a “Precision Rehabilitation” framework for dysphagia management in HNC as our analytical lens and organizing principle. As illustrated in **Figure 1**, this framework conceptualizes rehabilitation not as a static prescription, but as a dynamic, adaptive process initiated by comprehensive assessment and risk stratification, followed by personalized interventions that are continuously refined through monitoring and feedback. This model serves to explain inconsistencies in the existing literature and provides a structured framework for discussing how integrating patient-specific factors, treatment parameters, and behavioral support can yield improved, more consistent functional outcomes. The subsequent sections will evaluate the evidence supporting each component of this framework: from controversies in intervention timing and modality (Section 2) to the roles of radiotherapy dose (Section 3), comorbid conditions (Section 4), and advanced assessment tools (Section 5).

## 2 Swallowing rehabilitation: from evidence to controversy

Despite a growing body of research, the translation of swallowing rehabilitation evidence into consistent clinical benefit remains challenging, with ongoing debates centering on the optimal timing, modality, and implementation of interventions.

### 2.1 Preventive vs. therapeutic exercise: the timing debate

The question of when to initiate rehabilitation is pivotal, with approaches split between early preventive and late therapeutic strategies (27). The evolution of evidence reflects a growing understanding of this timing dilemma. An early prospective study by Carmignani et al. (2018) (3) provided initial support for the preventive approach, demonstrating that a program initiated before radiotherapy could lead to significant improvements in swallowing-related quality of life (MDADI) compared to standard care alone (3). This foundational work highlighted the potential of prehabilitation to modulate outcomes, but this study had a small sample size (n=68) and a short follow-up period of only 6 months. Subsequently, the ReDyor study (Guillen-Sola et al., 2019; 2025) (28, 29) directly compared early versus late initiation through a randomized design. Its results nuanced the earlier findings, demonstrating that while both timing strategies were feasible, neither was universally superior. Early initiation (before radiotherapy) was associated with better preservation of mouth opening at the end of treatment, whereas the late intervention group (after radiotherapy) showed significant recovery in expiratory muscle strength at the final assessment (29, 30). This suggested that the “best” time may be goal-oriented, dependent on the specific functional outcome targeted.

A major multicenter RCT by Hajdú et al. (2022) (7) further complicated the picture. This large trial (n=245) investigated a comprehensive intervention (swallowing exercises combined with progressive resistance training) administered during radiotherapy. While it did not demonstrate improvement in the primary outcome of swallowing safety, it revealed crucial benefits of the combined intervention through long-term follow-up data. Notably, the study’s large sample size and 12-month follow-up lend higher credibility to its conclusions.

**Figure 2:**
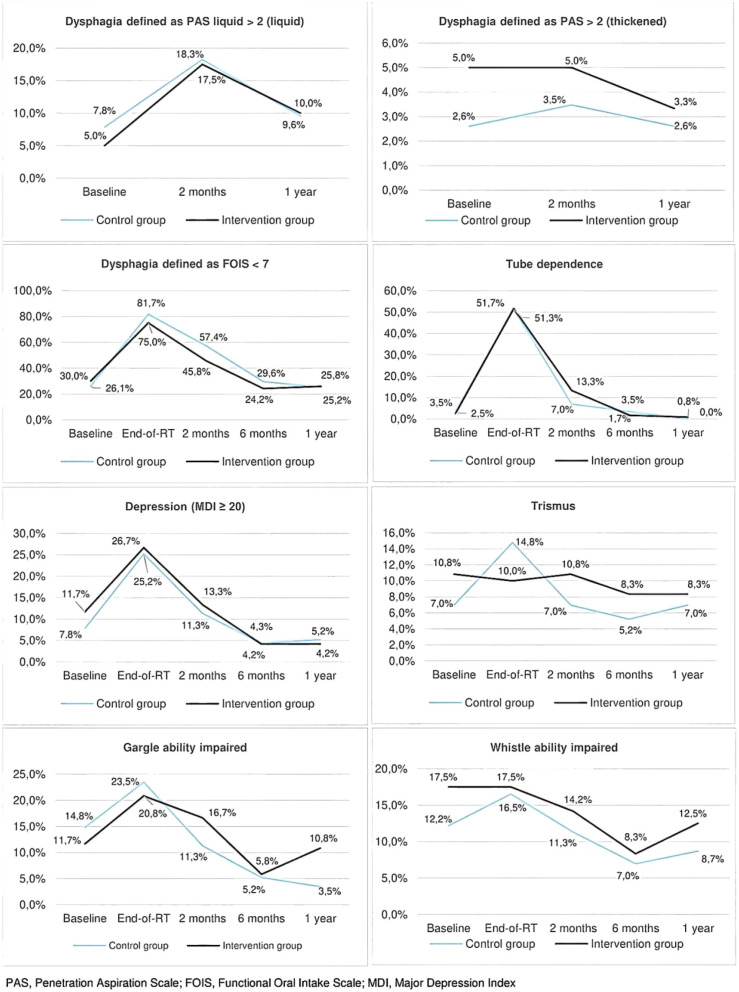
Long-term symptom trajectories in the Hajdú et al. RCT [7] comparing a combined swallowing exercise and progressive resistance training intervention versus standard care. Dynamic comparison of the incidence rates of six symptom/functional outcomes (such as dysphagia and trismus). Adapted from Hajdú S F, Wessel I, Dalton S O, et al. Swallowing Exercise During Head and Neck Cancer Treatment: Results of a Randomized Trial [J]. Dysphagia, 2021, 37 (4):749-762. https://doi.org/10.1007/ s00455-021-10320-5 [7]).

As illustrated in **Figure 2**, the intervention group consistently showed lower symptom incidence and faster recovery across multiple secondary outcomes. Notably, at 12 months, the intervention group’s incidence of liquid/viscous liquid dysphagia (12%/9%) was nearly half that of controls (21%/18%), demonstrating effective mitigation of persistent swallowing risks. The intervention also significantly reduced long-term complications, with trismus incidence at 4.2% versus 8.3% in controls, and improved psychological outcomes, as depressive symptoms decreased to 9% compared to 18% in controls.

These multidimensional benefits establish the value of radiotherapy-period intervention beyond the conventional “prevention versus treatment” timing debate. The findings support a goal-oriented approach to timing selection, where radiotherapy-period intervention proves particularly advantageous for achieving long-term functional preservation and psychological well-being, while short-term oral function maintenance may require additional stratification based on baseline patient characteristics like frailty status.

Most recently, the RCT by Petersson et al. (2025) (8) tested a simplified preventive exercise regimen during radiotherapy and found no significant effect on swallowing function or trismus at the end of treatment compared to usual care (8). However, a secondary analysis suggested a potential dose-response relationship, as patients with higher exercise adherence showed a non-significant trend towards better outcomes, underscoring the critical confounding role of adherence in interpreting timing studies.

In summary, as detailed in **Table 1**, the evidence for both timing strategies remains of moderate quality but points to distinct benefit profiles. The collective findings suggest a shift from a rigid “early vs. late” debate towards a more personalized, goal-oriented paradigm. Furthermore, the inconsistent findings across timing studies likely reflect the unaccounted-for heterogeneity in patient profiles, such as pre-treatment frailty (9), and the variable impact of adherence (17), underscoring the need for a more nuanced, stratified approach rather than a universal timing rule.

**Table 1:** Evidence summary and evaluation of swallowing rehabilitation interventions.

Intervention category	Specific measures	Level of evidence	Primary benefits	Limitations/controversies
Timing	Early Preventive Exercises	Moderate (28, 29)	May preserve mouth opening, respiratory muscle function	Long-term advantage unclear; optimal patient population undefined
	Late Therapeutic Exercises	Moderate (3)	Improves swallowing function and quality of life	May miss the critical window for functional preservation
Exercise Type	Traditional Maneuvers (e.g., Masako)	Moderate (5, 6)	Strong theoretical basis, widely applicable	Lack of standardization; limited evidence for efficacy in isolation
	Device-Assisted (e.g., SEA)	Low to Moderate (31)	Effectively increases muscle strength and volume; high feasibility	Evidence only in healthy elders; clinical outcomes unknown
	Combined Intervention (e.g., Voice Training)	Moderate (32, 33)	Synergistic improvement in swallowing and voice; reduces aspiration	Mechanism unclear; lacks combined assessment tools
	Neuromuscular Electrical Stimulation	Moderate (21)	Short-term pharyngeal improvement may surpass exercise alone	Device-dependent; long-term effects not superior
Adherence Strategies	Therapist Face-to-Face Support	High (17, 25)	Significantly improves adherence and functional outcomes	Resource-intensive, high cost
	Theory-Based Self-Management	High (26)	Improves adherence and emotional coping skills	Requires training, complex to implement
	mHealth/Digital Tools	Moderate (34)	High patient acceptance; facilitates remote monitoring	Gamification effects inconsistent (11)

Regarding the controversies and limitations, a primary barrier to resolving this debate is the lack of clear patient stratification criteria. Pre-treatment factors such as frailty have emerged as strong predictors of poor swallowing outcomes, suggesting a promising strategy of tailoring intervention timing based on individual risk profiles (e.g., initiating prehabilitation in high-risk frail patients) (23). However, this approach remains under-investigated in prospective trials. Furthermore, the field is constrained by studies with small sample sizes, short follow-up periods inadequate for assessing long-term radiation fibrosis, and significant heterogeneity in interventions and outcome measures, which complicates direct comparison and meta-analysis (3, 8, 28, 29).

### 2.2 Training modalities and techniques: What works best?

The landscape of swallowing rehabilitation has evolved from traditional maneuvers to incorporate increasingly sophisticated modalities (30, 35). The 2016 Cochrane review by Perry et al. (6) provided an early critical appraisal, concluding that evidence was insufficient to determine the benefit of therapeutic exercises over usual care, citing low-quality evidence and high heterogeneity among existing studies (5, 6). This highlighted the need for more rigorous and standardized research.

Subsequent research focused on developing and testing specific modalities. Kraaijenga SA group (2015) (31) conducted one of the first feasibility studies on a novel Swallowing Exercise Aid (SEA), demonstrating its capacity to significantly increase swallowing muscle strength and volume in healthy seniors (31). This pioneering work established the feasibility of device-assisted resistance training for swallowing musculature, though its applicability to HNC patients remained to be proven.

More recent studies have explored synergistic and combined approaches. Liu et al. (2024) (32, 33, 36) demonstrated in an RCT that adding the ABCLOVE voice training program to standard swallowing exercises resulted in significantly better swallowing function (SSA score), longer maximum phonation time, and lower rates of malnutrition and aspiration compared to swallowing exercises alone (32, 33, 36). This suggested a potent cross-system effect where phonatory exercises confer benefits to swallow physiology.

Concurrently, research into physical modalities has advanced. Ku et al. (2023) (14) conducted an RCT comparing transcutaneous neuromuscular electrical stimulation (TNMES) to traditional exercise-based swallowing training (EBST) in nasopharyngeal carcinoma patients. They found that TNMES yielded superior outcomes in improving pharyngeal function and quality of life in the short term, offering an effective alternative for specific patient populations (14).

As presented in **Table 1**, the evidence base for various modalities is expanding but remains graded from low to moderate. The most promising innovation is the shift toward combined and synergistic interventions that target multiple physiological systems (37). However, the field continues to be hampered by a lack of standardization in techniques, intensities, and durations. This heterogeneity prevents the isolation of active intervention components, complicates meta-analysis, and ultimately delays the formulation of definitive clinical guidelines. The optimal modality is therefore not only a question of exercise type but is also contingent upon individual patient factors, including the specific physiological deficits [e.g., as identified by instrumental assessment (38, 39)], and the patient’s ability and willingness to adhere to the prescribed regimen (26).

### 2.3 Adherence: the central challenge in rehabilitation

The critical importance of adherence has been increasingly recognized over the past decade. Early work, such as the review by Wells & King (2017) (40), systematically outlined the multifactorial nature of this problem, identifying key barriers including treatment burden, low motivation for preventive exercises, and information overload (40). This established a framework for understanding the adherence challenge.

The PRESTO trial (Baudelet et al., 2023) (25) marked a significant advance by directly comparing different service-delivery modes in a multicenter RCT. It provided high-quality evidence that while the mode of delivery (therapist-led vs. app-based vs. diary) significantly impacted adherence rates, the overall adherence level was a more critical determinant of functional improvement than the specific delivery method (17, 25). This underscored that ensuring high engagement is paramount, regardless of the channel.

Building on behavioral science, the PREPARE trial (Shinn et al., 2024) (26) demonstrated that a structured self-management intervention, incorporating principles of behavior change, could significantly improve adherence compared to standard follow-up. A key mechanistic finding was that improved emotional coping skills mediated 24% of the intervention’s effect, highlighting the psychological dimension of adherence (26).

Most recently, Charters et al. (2024) (17, 18) published a systematic review confirming that adherence is influenced by a complex matrix of factors, with regular clinical contact and social support being key facilitators, and radiotherapy toxicity being a primary barrier (17, 18). Concurrently, Shinn et al. (2024) (34) explored the frontier of digital health, testing a wearable swallowing activity sensor and reporting high patient acceptance for long-term monitoring, pointing to future technology-driven solutions.

In conclusion, research has progressively shifted from merely documenting poor adherence to actively testing multifaceted solutions. As **Table 1** shows, high-quality evidence now supports the effectiveness of theory-based self-management and frequent therapist contact. The key insight is that how patients are supported behaviorally and emotionally is as crucial as what exercises they are prescribed. The future lies in systematically embedding behavioral change techniques and digital tools into rehabilitation protocols to enable sustained adherence in real-world settings.

## 3 Radiotherapy dose and swallowing structures: from dose constraint to functional preservation

The refinement of radiotherapy techniques, particularly intensity-modulated radiotherapy (IMRT), has initiated a paradigm shift from solely maximizing tumor control to proactively preserving functional outcomes (41, 42). Understanding the precise dose-response relationships between specific swallowing structures and functional impairment is central to this endeavor, representing an area of both significant progress and ongoing debate. **Table 2** shows the summary of dose-effect relationships for swallowing-related structures.

**Table 2:** Summary of dose-effect relationships for swallowing-related structures.

Anatomical structure	Associated swallowing impairment	Key dosimetric parameters (examples)	Level of evidence	Clinical recommendation
Pharyngeal Constrictors (Middle/Inferior)	Acute & Late Dysphagia	Middle PC Dmean ≥50Gy; Inferior PC V55 ≥X% (13, 27); V50 < 50% (43)	High	Should be prioritized for constraint in IMRT planning
Suprahyoid Muscle Complex	Chronic RAD, Aspiration Risk	V69 (11)	High	Emerging key structure, especially in elderly patients
Geniohyoid Muscle	Impaired Hyoid Kinematics, Aspiration	Mean Dose & Min Dose (10)	Moderate	Strongly associated with swallow kinematics
Cricopharyngeus/Cervical Esophagus	Acute Dysphagia, PEG Dependency	Mean Dose (12)	Moderate	Key predictor for acute toxicity
Tongue Base/Tongue Muscles	Reduced Tongue Pressure, Oral Phase Impairment	To be further defined (39)	Low to Moderate	Important, but lacks unified dose threshold
Oral Cavity (Tongue Base, Hard Palate)	Chewing Dysfunction, Secondary Swallow Impairment	V40 < 30% (44)	Moderate	Consider for sparing to optimize long-term swallowing

### 3.1 Identification of critical structures and ongoing debates

The evolution of dose constraint targets demonstrates a clear trajectory from broad anatomical regions to precisely defined functional musculature. Early research established the foundational role of the pharyngeal constrictor muscles (PCs).The Langendijk group’s pioneering research first quantified the association between pharyngeal constrictor irradiation (>60 Gy) and long-term dysphagia, with their subsequent establishment of the V50 < 50% dose constraint for the PC complex (43, 45–47)—findings validated by the QUANTEC reports and now standard in IMRT planning. Building on this, Mazzola et al. (2014) (32) conducted a pivotal analysis revealing that specific dose parameters for the middle PC (Dmean ≥50Gy) significantly increased the risk of acute dysphagia. This was later reinforced by Mogadas et al. (2020) (13), who confirmed that doses ≥55Gy to the middle and inferior PCs were robustly associated with late dysphagia, establishing these muscles as high-priority structures for constraint in IMRT planning.

Concurrently, evidence emerged linking radiation dose to specific physiological mechanisms of impairment. Starmer et al. (2015) (23) provided a crucial kinematic correlation, demonstrating that radiation dose to the geniohyoid muscle was significantly associated with impaired hyoid bone kinematics and elevated aspiration risk. This established a direct link between dose to a specific suprahyoid muscle and functional swallowing impairment, providing a mechanistic explanation for its moderate evidence grade and strong association with kinematics (**Table 2**). A paradigm-shifting contribution came from the MD Anderson Head and Neck Cancer Symptom Working Group (2016) (11). Their large-scale analysis identified the suprahyoid muscle complex (with a specific threshold of V69) as a key predictor of chronic radiation-associated dysphagia, potentially surpassing the predictive power of the PCs for certain endpoints. This highlighted an emerging critical structure, particularly in elderly patients, and warranted a high evidence grade. Further refining our physiological understanding, Schaen-Heacock et al. (39) employed high-resolution manometry to objectively quantify swallowing pressures. Their work implicated the tongue base and related muscles in generating pressure deficits, explaining oral-phase impairment, though the specific dose thresholds for these structures remain to be definitively established, resulting in a lower current evidence grade. Alterio et al. (12) contributed to the focus on acute toxicity by identifying the cricopharyngeus and cervical esophagus as key structures. Their finding that the mean dose to these areas was a significant predictor for acute dysphagia and PEG dependency supports their classification as a moderate evidence grade predictor for acute toxicity. Notably, the Langendijk group’s 2011 research (44) expanded organ-at-risk (OAR) sparing beyond traditional structures to include the oral cavity (e.g., tongue base, hard palate), demonstrating that limiting V40 < 30% to these regions reduces long-term chewing dysfunction and subsequent swallowing impairment. This advancement aligns with our precision rehabilitation framework, reinforcing the value of personalized dose constraints tailored to individual anatomical profiles.

In summary, the identification of critical structures has evolved from a primary focus on the pharyngeal constrictors to include the suprahyoid complex, geniohyoid, cricopharyngeal region, and oral cavity components, each with varying levels of evidence supporting their role in swallowing impairment. The main controversy stems from the lack of a single “most critical” structure, as their importance appears context-dependent on the clinical endpoint and patient population.

### 3.2 The clinical value of dysphagia-optimized IMRT and proton therapy

The culmination of this dose-response research is the strategic implementation of Dysphagia-Optimized IMRT (DO-IMRT). The landmark phase III DARS trial (1) provided the first high-level evidence for this approach. This multicenter randomized controlled trial demonstrated that patients receiving DO-IMRT—which proactively applied dose constraints to swallowing structures like the pharyngeal constrictors—had significantly better swallowing-related quality of life at 12 months compared to those receiving standard IMRT, without compromising tumor control.

Proton therapy offers unique advantages over photon-based IMRT (including DO-IMRT) due to its Bragg peak characteristic, minimizing integral dose to non-target swallowing structures (e.g., suprahyoid muscles, cervical esophagus) (45, 48–50). Arnaud Beddok, et al. (48) reported that proton therapy was associated with a 30% lower rate of moderate-to-severe dysphagia (FOIS ≤5) at 12 months in HPV+OPC patients, with no compromise in tumor control. However, its accessibility remains limited by high costs, restricting its use to high-risk populations (e.g., large tumors requiring high RT doses near critical swallowing structures) (49, 50).

Overall, the DARS trial validates the clinical principle of proactively sparing functional swallowing structures, a strategy directly informed by the dose-response data summarized in **Table 2**. The addition of proton therapy and expanded OAR sparing (per Langendijk et al.) further enriches this approach (45). However, the generalizability of specific constraints is moderated by the DARS trial’s predominant enrollment of HPV-positive oropharyngeal cancer patients. Questions regarding long-term durability beyond 24 months, applicability to non-oropharyngeal HNC subsites, and the development of efficient clinical workflows for consistent contouring of these structures remain active areas of investigation.

## 4 Special populations and comorbidity management

The heterogeneity in functional outcomes following HNC treatment is not solely explained by tumor characteristics or treatment modality. A “precision rehabilitation” framework necessitates the recognition and management of specific patient phenotypes and comorbidities that significantly modulate the risk and severity of dysphagia.

### 4.1 The frail patient

Emerging evidence positions pre-treatment frailty as a powerful, independent predictor of poor swallowing outcomes (10, 13). A retrospective analysis of 242 patients demonstrated that those identified as frail prior to treatment had significantly worse swallowing-related quality of life and higher swallowing toxicity scores at 3, 6, and 24 months post-treatment compared to their non-frail counterparts (10). This suggests that frailty contributes to a diminished physiologic reserve, impairing the patient’s ability to withstand the functional insult of cancer therapy. These findings underscore the critical need for early identification of frailty using standardized geriatric assessment tools (e.g., G8, GFI), which should trigger proactive, tailored supportive care and rehabilitation strategies designed for this vulnerable population (10).

Patients undergoing postoperative adjuvant radiotherapy differ significantly in functional baselines, anatomical structures, and rehabilitation needs from those receiving radical chemoradiotherapy (51–53). Notably, a prospective study demonstrated that implementing swallowing rehabilitation during the postoperative adjuvant radiotherapy period was associated with significantly improved preservation of swallowing function in this patient cohort (51). This finding underscores the need for rehabilitation pathways tailored to the unique individual needs of this specific subgroup, rather than adopting a one-size-fits-all approach.

### 4.2 Lymphedema and swallowing function

The impact of head and neck lymphedema (HNL) on swallowing has been historically underappreciated. A comprehensive cross-sectional study revealed that 99% of patients post-treatment had some form of HNL (internal or external) (4). Crucially, the severity of both internal and external lymphedema was independently associated with worse outcomes across multiple domains: it correlated with an increased risk of penetration/aspiration, greater dietary restrictions, and a higher burden of patient-reported swallowing symptoms (4, 6). This establishes HNL not merely as a cosmetic issue but as a direct contributor to swallowing pathophysiology. Consequently, routine clinical assessment for dysphagia must be expanded to include systematic evaluation of lymphedema, as its management may be integral to restoring safe and efficient swallowing (4, 22).

### 4.3 HPV-positive oropharyngeal cancer patients undergoing primary surgery

HPV-positive oropharyngeal cancer (HPV+OPC) is a distinct subgroup with evolving treatment approaches, including increasing use of primary surgery (e.g., transoral robotic surgery [TORS], radical neck dissection) followed by adjuvant radiotherapy (RT) for select patients (48, 54). This population faces unique swallowing rehabilitation challenges, as surgical anatomical changes and adjuvant RT-induced toxicity impose a “double burden” on swallowing function. Surgical resection modifies pharyngeal structure, sensory feedback, and airway protection—TORS may disrupt pharyngeal constrictor integrity, while radical neck dissection impairs neuromuscular control of swallowing musculature (52, 54). Adjuvant RT exacerbates these deficits via tissue fibrosis, mucosal inflammation, and xerostomia, increasing aspiration risk, dietary restrictions, and long-term feeding tube dependency compared to primary RT alone (48). Recent evidence supports targeted rehabilitation: initiating swallowing exercises (e.g., Shaker maneuver, tongue-press) pre-adjuvant RT preserves pharyngeal function and sensory-motor integration, improving Functional Oral Intake Scale (FOIS) scores and reducing aspiration (52). Combining swallowing training with expiratory muscle strength training (EMST) and saliva substitutes enhances oral intake tolerance and reduces mucositis-related pain (48), while patients with extensive soft palate/base-of-tongue resection require intensified mucosal protection and sensory re-education (55).

Rehabilitation timing is critical—pre-adjuvant RT initiation capitalizes on pre-surgical reserve, minimizing irreversible fibrosis (46). Individualization should account for surgical extent, RT dose to swallowing structures (e.g., suprahyoid muscle complex V69), and pre-treatment frailty (48, 54, 55). Interdisciplinary teams (speech-language pathologists, surgical/radiation oncologists) are essential to tailor plans, with landmark studies (48, 49, 52, 55) providing a robust evidence base for this growing subgroup.

### 4.4 Trismus

Trismus (severely restricted mouth opening) is a highly prevalent and persistent complication, with meta-analyses indicating a peak prevalence of 44.1% at 6 months post-radiotherapy, persisting in about one-third of survivors in the long term (56, 57). The functional consequences for oral intake and hygiene are profound. While exercises are the cornerstone of management, their effectiveness is often limited, particularly during the acute toxicity phase of radiotherapy (41, 58, 59). This has spurred the development of mechanical devices, such as the Therabite^®^ system and the novel EZBite device. Studies suggest that these devices can be effective, with EZBite demonstrating significant improvements in maximum interincisal opening (MIO) and trismus-related quality of life compared to conventional exercises alone (15). The current evidence supports a multimodal approach that combines custom-fitted mechanical stretching devices with guided exercise regimens to maximize outcomes (15, 59).

### 4.5 Oral health and swallowing: impact of impaired dentition

Oral health, particularly dentition status, is a critical but underrecognized factor affecting swallowing function in HNC survivors. Edentulism or impaired dentition (e.g., partial tooth loss) significantly impacts swallowing safety and requires integration into rehabilitation planning (60–62). Mechanistically, poor dentition impairs chewing, leading to larger food boluses, prolonged pharyngeal transit, and higher aspiration risk—risks amplified by RT-induced pharyngeal weakness (61). The prevalence of dentition loss in HNC survivors is alarmingly high, with 40–60% developing tooth loss post-treatment (61, 62). Key risk factors include RT-induced xerostomia (reducing salivary buffering and increasing dental caries), osteoradionecrosis of the jaw (limiting dental retention), and pre-treatment poor oral health—factors that are often compounded by long-term dietary restrictions and reduced oral hygiene compliance post-RT. From a rehabilitation perspective, integrating dental assessment and intervention into the precision rehabilitation framework is essential.

We recommend incorporating prosthodontic evaluation into pre-RT comprehensive assessments (detailed in Section 5) to identify pre-existing dentition issues and plan proactive interventions (e.g., dental prosthetics, preventive fluoride treatments). Post-treatment, swallowing exercises should be paired with oral health interventions, such as saliva substitutes (alleviating xerostomia) and dental prosthetic fitting (improving chewing efficiency), to optimize bolus preparation and enhance swallowing safety (62). Emerging evidence (61, 62) confirms that dentition status is an independent predictor of swallowing outcomes in HNC survivors, highlighting the clinical value of this integrated approach.

## 5 Advancements in assessment tools and the need for standardization

Within the precision rehabilitation framework for HNC dysphagia (**Figure 1****),** accurate and comprehensive assessment forms the critical foundation for risk stratification and personalized intervention. As illustrated in the conceptual framework, the pathway begins with Pretreatment Frailty evaluation and moves through Comprehensive Assessment, which directly informs Risk Stratification into low, medium, and high-risk categories. Recent advancements in both patient-reported outcome measures (PROMs) and objective instrumental tools have enabled this increasingly sophisticated, multi-modal evaluation paradigm.

The evolution of subjective assessment has been marked by cross-cultural validation efforts, particularly with the M.D. Anderson Dysphagia Inventory (MDADI). Its rigorous translation and validation into multiple languages have demonstrated excellent reliability, facilitating international multi-center trials and ensuring accurate capture of patient perspectives across populations (63). This standardization is essential for generating generalizable evidence, directly supporting the Patient-Reported Outcomes (PROMs) component within the framework’s assessment module.

Simultaneously, objective instrumental assessments have advanced significantly, with FEES/VFSS now incorporating validated scoring systems like DIGEST-FEES (64). These tools provide the anatomical and kinematic profiling essential for initial risk stratification and subsequent Instrumental Assessments shown in the framework. Furthermore, high-resolution manometry (HRM) has emerged as a powerful technology that quantifies pharyngeal swallowing pressures, revealing specific impairment patterns invisible to traditional imaging (39). This detailed physiological profiling enables the Continuous Monitoring & Dynamic Adjustment central to the adaptive rehabilitation process.

The framework emphasizes the necessity of multi-modal assessment, where a comprehensive evaluation integrates (65–67): (1) Patient-reported outcomes (e.g., MDADI) capturing functional impact; (2) Clinical/functional scales (e.g., FOIS) documenting dietary status; (3) Instrumental visualization (e.g., VFSS/FEES) identifying pathophysiology; (4) Physiological quantification (e.g., HRM) measuring biomechanical deficits. This integrated approach, feeding into the framework’s Feedback Loop for Intervention Adjustment, moves beyond merely detecting aspiration to understanding its underlying mechanisms—essential for tailoring interventions in precision rehabilitation (67).

Despite these advancements, the lack of universal standardization in assessment protocols remains a challenge, hindering cross-study comparisons and meta-analyses. Future efforts must focus on establishing core outcome sets and standardized assessment batteries that align with the framework’s comprehensive approach, particularly supporting the Dynamic Adjustment processes that ensure rehabilitation remains responsive to patient progress throughout the care continuum.

## 6 Future directions: a translational roadmap for precision rehabilitation

The evidence synthesized in this review compellingly argues for a paradigm shift from one-size-fits-all regimens to the dynamic, precision rehabilitation framework introduced in **Figure 1**. However, conceptual models must be translated into actionable strategies. This section outlines a detailed translational roadmap, identifying key research priorities and methodological innovations required to bridge the enduring evidence-to-practice gap and make precision rehabilitation a clinical reality.

### 6.1 Deconstructing and quantifying the “black box” of interventions

A fundamental barrier to personalization is the persistent “black box” nature of many swallowing interventions. Future research must prioritize the mechanistic deconstruction of common therapies.

Example Research Questions: Does the Masako maneuver primarily strengthen the base of tongue, improve pharyngeal wall contraction, or facilitate earlier laryngeal vestibule closure? For which specific physiological deficit (e.g., reduced tongue base retraction identified on HRM) is it most indicated?

Methodological Pathway: Employ HRM and dynamic MRI concurrently with standardized interventions in well-defined patient subgroups. This will allow researchers to link specific exercise components to quantifiable changes in biomechanics (e.g., pressure generation, hyoid displacement). The goal is to create a “menu” of evidence-based exercises, each mapped to a specific physiological target, enabling clinicians to prescribe based on a patient’s unique impairment profile rather than a generic diagnosis of “dysphagia.”

### 6.2 Next-generation risk stratification: integrating multimodal data with AI

The framework’s initial risk stratification must evolve from relying on single factors (e.g., frailty or mean pharyngeal constrictor dose) to integrating multifaceted data streams (68). This evolutionary direction is aligned with the latest research by Langendijk JA’s group (2025), which has focused on the pivotal contributions of deep learning models to enhance risk prediction accuracy (60).

Technically, the key lies in developing machine learning (ML) models that fuse structured data—such as tumor stage, pre-treatment MD Anderson Dysphagia Inventory (MDADI) scores, and frailty index—with unstructured or complex data modalities. These include dosiomics [extracting advanced texture and shape features from the 3D dose distribution of swallowing structures, which may outperform simple mean dose in predictiveness (11)], radiomics (analyzing pre-treatment CT or MRI scans of swallowing muscles to quantify density or texture features indicative of radiation damage susceptibility), and patient genomics (exploring fibrosis-related genetic polymorphisms, e.g., TGF-β pathway genes, to identify high-risk patients for late fibrotic dysphagia).

Clinically, Clinically, the output of such integrated ML models would be a validated, continuously learning predictive algorithm. This algorithm will assign each patient a personalized risk score at the start of treatment and recommend a corresponding rehabilitation pathway intensity—such as targeted segmental tongue function training for patients with predicted tongue pressure deficits (69) or combined swallowing training with feeding management for those undergoing particle therapy (70)—thereby translating multifaceted data into actionable clinical guidance for precision rehabilitation. Notably, evidence-based swallowing facilitation strategies (e.g., prophylactic training paired with feeding management) have been rigorously designed to align with such personalized pathways, further supporting the framework’s clinical applicability (36).

### 6.3 Technology-enabled closed-loop rehabilitation

The “Monitoring & Feedback” loop in **Figure 1** can be automated and optimized through technology, creating a closed-loop system that adapts to patient progress in near real-time.

Wearable Sensors for Adherence and Efficacy: Beyond the initial prototypes (34), next-generation sensors should validate their ability not only to count swallows but also to qualitatively assess swallow effort or efficiency. Coupling these sensors with a smartphone app that uses computer vision to provide real-time feedback on exercise performance (e.g., correct laryngeal elevation during a Shaker exercise) could transform home-based practice. Such tools are particularly relevant given the feasibility of structured home exercise programs, including multimodal self-help interventions that combine speech, swallowing, and shoulder training (71), as well as combined swallowing therapy with progressive resistance training (PRT) (72).

AI-Powered Instrumental Assessment: Software tools that use deep learning to automatically analyze VFSS or FEES videos are urgently needed. These tools should objectively measure key kinematic parameters (e.g., hyoid bone trajectory, Penetration-Aspiration Scale score) and generate standardized reports, freeing up clinician time and eliminating inter-rater variability, which is a major confounder in current research and practice (64).

Adaptive Intervention Platforms: Develop mHealth platforms that function as “digital coaches.” Based on data from sensors and patient-reported symptoms, these platforms could automatically adjust exercise difficulty (e.g., increasing repetition count), provide motivational messaging, and flag patients who are non-adherent or deteriorating for early clinician intervention. Online self-care education programs have already demonstrated feasibility and high patient satisfaction among post-laryngectomy survivors (73), laying the groundwork for scalable adaptive platforms.

### 6.4 Implementation roadblocks and mitigation strategies

Despite the theoretical innovation of the proposed precision rehabilitation framework, its clinical translation faces multiple practical barriers. Targeted mitigation strategies are as follows: For cost and resource constraints, a ‘phased implementation’ model is recommended—primary phase (resource-constrained institutions) focuses on core components (e.g., frailty screening using the G8 questionnaire (10), personalized swallowing exercise prescriptions) such as guided home-based prophylactic exercise programs (74), without the immediate need for AI or wearable devices; advanced phase (tertiary hospitals/cancer centers) can scale the deployment of remote monitoring tools (26) through partnerships with digital health companies to reduce technology access costs. For clinician training: Develop standardized, concise training modules (duration ≤4 hours) covering ‘use of frailty screening tools,’ ‘interpretation of core framework processes,’ and ‘key points of interdisciplinary communication,’ supplemented by case manuals (including rehabilitation program examples for patients at different risk levels) to enhance training accessibility. For Interdisciplinary collaboration barriers: Embed an ‘automatic referral for swallowing rehabilitation’ function in electronic health record (EHR) systems—after radiation oncologists complete dose planning, the system automatically triggers referrals based on patient frailty status and radiotherapy dose parameters, connecting rehabilitation and nutrition teams to streamline collaboration workflows.

Furthermore, the adaptability of the framework is a core guarantee of its feasibility: clinicians can flexibly adjust intervention intensity and technology selection according to the resource level of their institutions and patient population characteristics (e.g., community hospitals with a high proportion of elderly frail patients), rather than enforcing ‘full-component implementation.’ Existing studies have confirmed that the implementation of core components alone—such as targeted intensive oropharyngeal training for nasopharyngeal carcinoma survivors (57)—can significantly improve patient outcomes (52), providing a practical basis for the gradual promotion of the framework.

To accelerate the translation of the precision rehabilitation framework into clinical practice, future implementation-focused research should prioritize three key areas: (1) Multicenter pilot studies (11) to test the feasibility of core framework components across medical institutions with varying resource levels, and quantify the cost-benefit ratio of resource inputs versus clinical outcomes (e.g., reduced aspiration pneumonia rates); (2) Reimbursement policy research to advocate for the inclusion of ‘precision rehabilitation assessments’ (e.g., HRM (39), frailty screening (10)) in medical insurance schemes, alleviating the economic burden on healthcare institutions and patients; (3) Qualitative surveys of clinician acceptance (17) to identify subjective barriers to framework adoption (e.g., cognitive load, operational complexity) and optimize implementation strategies accordingly.

### 6.5 Expanding the scope: addressing understudied populations and long-term outcomes

The evidence base remains skewed towards HPV-associated oropharyngeal cancer survivors. Precision frameworks must be validated across the entire HNC spectrum.

Target Populations: Non-Oropharyngeal Cancers: Develop and test specific rehabilitation pathways for patients with oral cavity, laryngeal, and hypopharyngeal cancers, whose functional deficits and surgical reconstructions present unique challenges.

The Elderly and Frail: Tailor interventions for the geriatric HNC population, focusing on interventions that can be performed seated, that account for multi-morbidity, and that prioritize functional independence and QoL over idealized physiological outcomes.

Long-Term Survivorship (>5 years): Initiate prospective, longitudinal cohort studies that track the natural history of dysphagia decades after treatment. This is critical for understanding and preventing late, progressive functional decline due to accelerated aging and fibrosis, an area currently shrouded in anecdote.

In conclusion, the path forward is both challenging and exhilarating. It requires a concerted, collaborative effort that bridges engineering, data science, behavioral psychology, and clinical medicine. By systematically addressing these research priorities—from deconstructing interventions and building intelligent risk models to creating adaptive digital health systems and tackling implementation science—we can transition the precision rehabilitation framework from a compelling conceptual diagram into a robust, scalable, and equitable standard of care that truly optimizes the functional future of every head and neck cancer survivor.

## 7 Conclusion

This study is a narrative review, and its inherent methodological limitation lies in not adopting the PRISMA workflow for exhaustive search as systematic reviews do. The selection strategy focusing on ‘high-impact studies’ may introduce selection bias. It is important to note that the core objective of this review is not to quantify intervention effect sizes, but to critically synthesize evidence, resolve field controversies, and propose a precision rehabilitation framework—an objective highly aligned with the strengths of narrative reviews in ‘in-depth interpretation + conceptual integration.’ Furthermore, we have contextualized our findings within the broader evidence base by cross-referencing published systematic reviews (e.g., Perry et al., 2016; Charters et al., 2024), thereby partially mitigating the impact of selection bias. Future studies could further validate the generalizability of this framework through systematic reviews and meta-analyses.

The management of dysphagia in head and neck cancer has decisively moved beyond the question of if rehabilitation is necessary, to the more complex and critical challenge of how to optimally deliver it. This review synthesizes evidence underscoring that the historical paradigm of uniform exercise prescriptions is inadequate to address the profound heterogeneity in patient presentations, treatment toxicities, and functional outcomes. The inconsistent efficacy of swallowing interventions, as highlighted in early systematic reviews (5, 6), is not a reflection of their inherent futility, but rather a consequence of this one-size-fits-all approach and the pervasive challenge of patient adherence (17, 19).

The path forward lies in the adoption of a Precision Rehabilitation framework. This model demands deep cross-disciplinary collaboration, integrating insights and techniques from radiation oncology [e.g., Dysphagia-Optimized IMRT (1), refined dose constraints for structures like the suprahyoid complex (11)], rehabilitation medicine [e.g., multimodal training (33), advanced instrumental assessment (32, 64, 68)], behavioral science [e.g., theory-based self-management (21, 26)], and digital health [e.g., wearable sensors for monitoring (34)]. It is at the intersection of these fields that meaningful progress will be made.

Future research must therefore pivot from asking broad efficacy questions to focusing on the development and validation of personalized, sustainable, and technology-assisted rehabilitation models. This entails creating risk-stratification tools that incorporate factors like pre-treatment frailty (23), designing adaptive interventions that respond to patient progress and barriers, and leveraging technology not merely as a reminder system but as an integral component of assessment, feedback, and engagement. By converging these strands, the field can finally bridge the evidence-to-practice gap, ensuring that advancements in cancer survival are matched by equally significant improvements in the long-term functional well-being and quality of life for every head and neck cancer survivor.
